# Malignant transformation of hepatic endometriosis: a case report and literature review

**DOI:** 10.1186/s12905-021-01366-6

**Published:** 2021-06-21

**Authors:** Dandan Wang, Qing Yang, Huaitao Wang, Chang Liu

**Affiliations:** 1 Department of Obstetrics and Gynecology, Shengjing Hospital of China Medical University, No. 36 Sanhao Street, Heping District, Shenyang, 110004 People’s Republic of China; 2 Department of Pancreas and Thyroid Surgery, Shengjing Hospital of China Medical University, No. 36 Sanhao Street, Heping District, Shenyang, 110004 People’s Republic of China; 3 Department of Pathology, Shengjing Hospital of China Medical University, No. 36 Sanhao Street, Heping District, Shenyang, 110004 China

**Keywords:** Extrapelvic endometriosis, Malignant transformation, Liver neoplasms, Adenocarcinoma

## Abstract

**Background:**

Extrapelvic endometriosis is defined as the presence of ectopic endometrial tissue in structures outside the pelvis. Although extra-pelvic endometriosis is generally considered benign conditions, malignant potential within endometriotic foci occurs even after definitive surgery. Malignant transformation of hepatic endometriosis is extremely rare. Preoperative diagnosis of this cancer is difficult, and no guidelines on the optimal management currently exist. Here, we present a case report of malignant transformation of hepatic endometriosis and a brief literature review to highlight the current knowledge of the prevalence, clinical features, diagnosis, and management of this condition.

**Case presentation:**

A 50-year-old woman with a 2-year duration of progressive right upper quadrant abdominal pain was admitted to the hospital. She underwent hysterectomy and bilateral salpingo-oophorectomy for benign conditions 4 years prior. Tumor markers demonstrated elevated carbohydrate antigen (CA)-199 112U/mL (normal range: 0–35U/mL) only. Radiological imaging suggested the presence of a 10.7 × 7.7-cm mass in the right lobe of the liver extending to the diaphragm. The intraoperative frozen sections suggested malignant tumor. Right hepatectomy with infiltrating diaphragm resection was performed. The final pathology with immunohistochemistry staining confirmed endometrioid adenocarcinoma in the liver originating from preexisting hepatic endometriosis. After the multidisciplinary team meeting, the consensus was surgery followed by adjuvant chemotherapy. To our knowledge, this is the first case of Chinese woman of a malignant liver tumor originating from endometriosis ever reported by reviewing the current English medical literature.

**Conclusion:**

Though rare, extrapelvic endometriosis-associated cancers should be considered as differentiated diagnosis even after hysterectomy and bilateral salpingo-oophorectomy. This case highlights the importance of collaborative efforts across multiple disciplines for accurate diagnosis and appropriate treatment of malignant transformation of hepatic endometriosis.

**Supplementary Information:**

The online version contains supplementary material available at 10.1186/s12905-021-01366-6.

## Background

Endometriosis, defined as the presence of endometrial glands and stroma outside the uterine cavity, affects 6 ~ 10% of reproductive-aged women [1] and 2 ~ 4% of postmenopausal women [2]. Endometriosis is most frequently located in pelvic organs. However, unusual and remote organs of involvement include the umbilicus, incisional scars, diaphragm, gastrointestinal tract, bladder, lungs, pancreas, liver, and heart have also been described [3]. Although both pelvic and extrapelvic endometriosis are generally considered benign conditions, malignant potential within endometriotic foci has been well documented [4]. The ovaries are the primary sites in 78.7% of all malignancies arising from endometriosis, extragonadal sites compose the remaining 21.3% [5].Malignant transformation of hepatic endometriosis is extremely rare, with only a few cases reported in the literature. Herein, we describe the case of a patient with endometrioid adenocarcinoma of the liver arising from endometriosis following a remote total abdominal hysterectomy and bilateral salpingo-oophorectomy (TAH/BSO).

## Case presentation

A 50-year-old woman with a body mass index 23.1 kg/m^2^ was referred to our hospital due to a 2-year history of recurrent right upper quadrant abdominal pain with progressive aggravation. Past medical history included a cesarean section at age 40 and TAH/BSO for atypical endometrial hyperplasia and ovarian endometrioma at age 46. The patient had not taken hormonal replacement therapy (HRT) since age 46. No family history of malignancy was recorded. Physical examination revealed right upper quadrant tenderness without any palpable masses. Fever, jaundice, vomiting, or other clinical signs were not observed. Liver function and viral serological tests for hepatitis B and C were normal. Tumor markers demonstrated an elevated carbohydrate antigen (CA)-199 level (112 U/mL [normal: 0–35 U/mL]); CA-125, carcinoembryonic antigen, and alpha fetoprotein levels were normal.

**Fig. 1:**
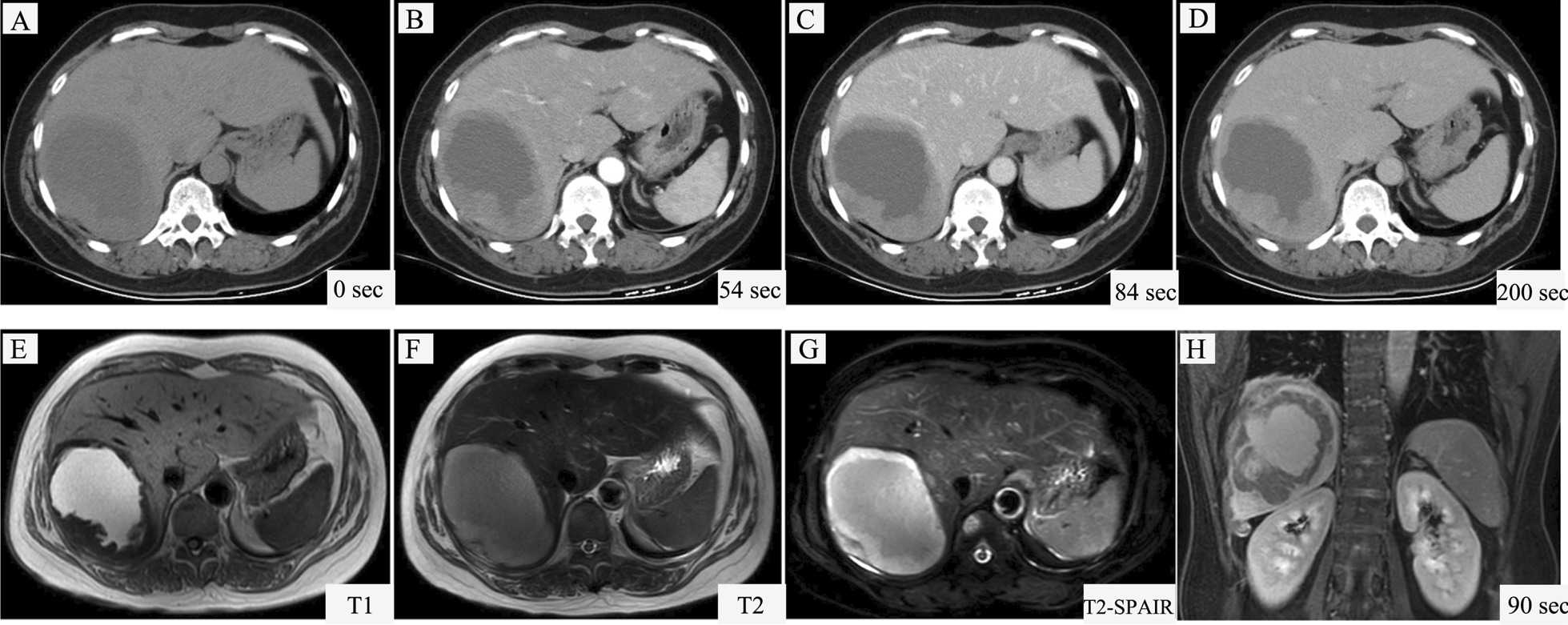
Imaging findings of the patient. Computed tomography (CT) scan showed an ill-defined, hypodense mass in the right liver lobe extending to the diaphragm (**A**) with moderate enhancement in the periphery of the mass at 54 s (**B**), 84 s (**C**) and 200 s (**D**) after intravenous injection of contrast. Magnetic resonance imaging (MRI) demonstrated an elliptic mass in the right liver lobe with an increased signal on both T1- (**E**) and T2- (**F**) images; On T2- SPAIR sequence the mass showed high signal indicating haemorrhage within the mass (**G**); **H** Post-contrast axial T1-weighted sequence showed the capsule of the right lobe of liver was unevenly thickened and enhanced but no obvious enhancement was visible inside the tumor

Contrast-enhanced computed tomography (CT) scan showed an ill-defined, hypodense mass of approximately 10.7 × 7.7 cm in the right liver lobe extending to the diaphragm (Fig. 1A), with moderate enhancement in the periphery during the arterial phase (Fig. 1B–D). Magnetic resonance imaging (MRI) of the liver demonstrated this lesion had an increased signal on both T1- (Fig. 1E) and T2- weighted images (Fig. 1F). No change was observed on T2-spectral presaturation attenuated inversion recovery (SPAIR) sequence (Fig. 1G), indicating hemorrhaging within the mass. The capsule of the right lobe of liver was unevenly thickened and enhanced, but no obvious enhancement was visible inside the tumor (Fig. 1H). No lymph node involvement was observed.

Laparotomy was performed, revealing a large complicated mass in segment VII. Multiple nodules on the diaphragm, ranging from 1 to 3 cm in diameter, were also observed. Some of the nodules were cystic with hemorrhagic contents and densely adhered to the surface of the liver. The abdominal cavity exploration revealed no other abnormalities. During adhesiolysis, the hepatic intraparenchymal mass ruptured, expelling large amounts of “fish flesh”-like tissue and hematoma. Some of the tissue and one diaphragmatic nodule were sent for intraoperative frozen sections, which suggested sporadic broken endometrial-like tissues. Part of the hepatic mass was resected for a second intraoperative frozen section, which unexpectedly indicated a malignancy. After fully communicating with the patient’s family, anatomical resection was performed, including right hepatectomy with infiltrating diaphragm resection. The patient had an uneventful postoperative recovery without complications.

**Fig. 2:**
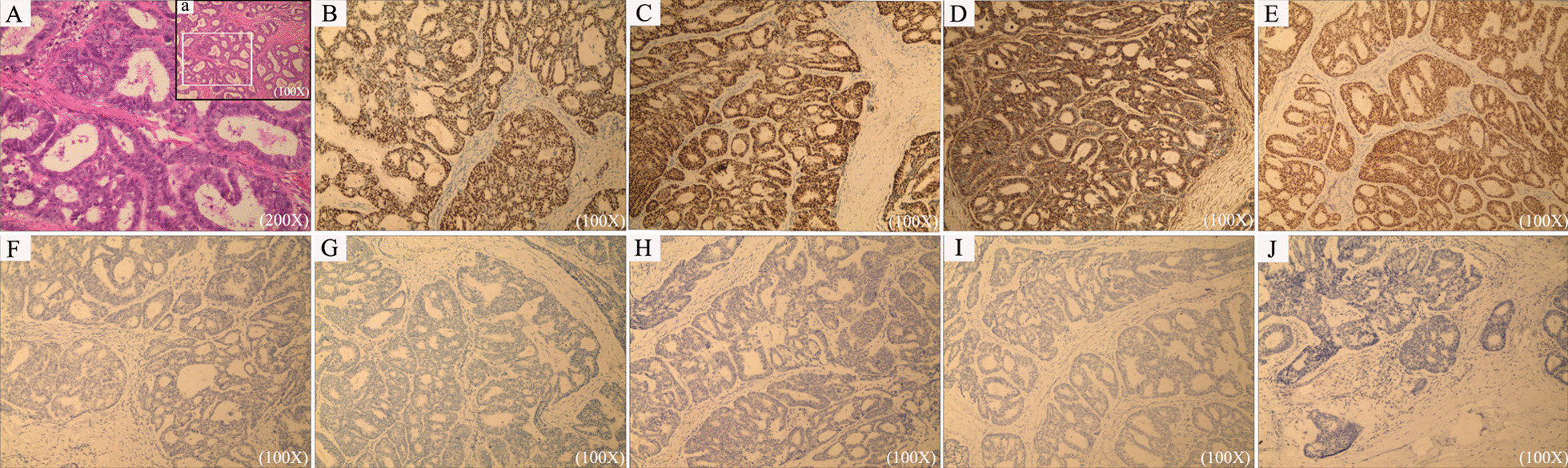
Histopathological and immunohistochemical staining findings. **A** Highly to moderately differentiated endometrioid adenocarcinoma, with tumor cells arranging in a glandular or sieve pattern with proliferative fibrous tissue surrounding (200 × ; H&E staining); **a** Highly to moderately differentiated endometrioid adenocarcinoma (100 × ; H&E staining), the white box area was magnified to **A**; **B** Positive stain for estrogen receptor (100 ×); **C** Positive stain for progesterone receptor (100 ×); **D** Positive stain for vimentin (100 ×); **E** Positive stain for paired-box gene 8 (100 ×); **F** Negative stain for cytokerin-7(100 ×); **G** Negative stain for arginase 1 (100 ×); **H** Negative stain for hepatocyte (100 ×); **I** Negative stain for calretinin (100 ×); **J** Negative stain for glypican-3 (100 ×)

Final histopathological examination of the surgical specimens confirmed the diagnosis of highly- to moderately-differentiated endometrioid adenocarcinoma in the liver. The tumor cells had pleomorphic nuclei and a high nucleus/cytoplasm ratio, and were arranged in a glandular or sieve pattern surrounded by proliferative fibrous tissue (Fig. 2A and a). Immunohistochemistry found that tumor cells were strongly positive for estrogen receptor (Fig. 2B), progesterone receptor (Fig. 2C), vimentin (Fig. 2D), paired-box gene -8 (Fig. 2E), but negative for cytokerin -7 (Fig. 2F), Arginase 1 (Fig. 2G), hepatocyte (Fig. 2H), calretinin (Fig. 2I), and glypican-3 (Fig. 2J). Although the specimen was sampled extensively, the classic triad of endometrial glands, stroma, and hemosiderin-laden macrophages was not identified.

The case was discussed at a multidisciplinary team meeting involving radiologists, pathologists, oncologists, and gynecologic surgeons. Clinical and immuno-histopathological findings strongly supported the diagnosis of endometrioid adenocarcinoma originating from preexisting hepatic endometriosis. Adjuvant chemotherapy was recommended for treatment. The patient agreed with the treatment regimen and had completed three courses of chemotherapy (doxorubicin combined with cis-platinum, every 3 weeks) with sufficient tolerance (Blood tests showed Grade I myelosuppression that could be reversed by oral drugs.) at the time of writing. Long-term follow-up will be conducted to observe the prognosis.

## Discussion and conclusion

Extrapelvic endometriosis is defined as the presence of ectopic endometrial tissue in structures outside the pelvis and has been described in almost all other remote organs of the human body such as the lungs, the brain, the urinary system, the gastrointestinal tract, the central nervous system, and the abdominal wall [3]. Hepatic endometriosis, first described by Finkel et al. in 1986 [6], is an extremely rare form of extrapelvic endometriosis, and its exact prevalence is not easily estimated due to the lack of population-based epidemiological trials [3]. In the current English literature, only 39 cases of hepatic endometriosis have been reported. An additional table shows the characteristics, presentation, and treatment of those cases in more detail (see Additional file 1: Table 1).

The etiology and pathogenesis of extrapelvic endometriosis remain unknown. Various hypotheses have been proposed; however, to date none of the hypothesized mechanisms was able to exhaustively explain every reported case [3]. The implantation theory suggests that endometrial fragments are transplanted into extra-pelvic sites through retrograde menstruation, lymphatic or hematogenous dissemination, or iatrogenic injury. An immune dysfunction that interferes with the cleaning of the ectopic endometrial fragments and provokes an inflammatory milieu is supposed to play a vital role during the formation and progression of endometriotic lesion [7, 8]. However, this theory does not explain the appearance of the disease in girls before or shortly after menarche, in women affected by Mayer–Rokitansky–Kuster–Hauser’s syndrome, or even in men [8]. The coelomic metaplasia theory and the Müllerian remnants hypothesis may explain the occurrence of endometriosis outside the pelvis [8]. The metaplastic transformation or differentiated changes are supposed to occur secondary to hormonal influences, inflammatory processes, (epi)genetic factors, immune alterations, and environmental factors [8]. Moreover, the involvement of stem cells originating from bone marrow or the basal layer of the endometrium opened a new scenario that shed light on the pathogenesis of both pelvic and extrapelvic endometriosis [9, 10]. Indeed, it has been demonstrated that they could spread through vessels or fallopian tubes and differentiate into endometrial tissue at different anatomic sites, perhaps because of a failure of the immune system [7, 8]. However, the heterogeneity of extrapelvic endometriosis and the different contexts in which it develops suggest that a single etiopathogenetic model is not sufficient to explain its complex pathobiology [8].

Malignant transformation of endometriosis, initially described by Sampson in 1925 [11], has been estimated to affect approximately 1% of premenopausal women suffering from endometriosis, and 1 ~ 2.5% of postmenopausal women, while the vast majority of them arising in the ovary [12]. Extragonadal malignant transformation is extremely rare phenomenon, but has been reported in distant sites or even after definitive surgery (TAH/BSO). To our knowledge, this is the first case of Chinese women of liver malignant tumor originating from endometriosis ever reported and there are another four cases in the English medical literature [13–16], summary provided in Table 1. As reviewed by Králíčková et al. [17], many studies have reported a consistent correlation between endometriosis and ovarian cancer according to histological subtypes. However, the question of how much higher the absolute risk is not fully clear. Nevertheless, due to rarity of the event, the existing evidence is insufficient to elucidate the absolute risk of malignant transformation of extrapelvic endometriosis.

**Table 1:** Main characteristics of reported cases of hepatic malignant tumors arising from endometriosis

Author; year	Age (y)	Menopausal state/HRT	Prior surgery	History of endometriosis	Primary symptom/physical examination	Preoperative diagnosis/lesion size/location	Treatment	Histological subtype	Adjuvant therapy	Follow-up (y)
Weinfeld [13]	60	Post/NA	HY + BSO; urinary bladder endometriosis resection	Yes	Right upper quadrant tenderness/NA	CT/3.1 cm/right lobe	Right diaphragmatic/perihepatic mass resection, left hepatectomy and cholecystectomy	Endometrioid adenosquamous carcinoma (moderately differentiated)	NA	NA
N‘Senda [14]	54	Post/Yes	HY + BSO	Yes	Right-sided epigastric pain/hard palpable mass with tenderness in the right hypochondra	CT, MR/20 cm/right lobe	Right hepatectomy with adjacent diaphragm	Adenosarcoma	No	2
Khan [15]	59	Post/NA	HY + BSO; laparotomy for intestinal endometrial stromal sarcoma (ESS)	Yes	Right upper quadrant abdominal pain/hepatomegaly	CT/NA/right lobe	Right hepatectomy	ESS (low-grade)	No	4
Knowles [16]	46	Post/NA	HY + BSO + appendectomy	Yes	NA	NA	Right trisegmentectomy radical bile duct excision + lymphadenectomy	Endometrioid adenocarcinoma No	No	1
Present case	50	Post/No	HY + BSO; cesarean section	Yes	Right upper quadrant abdominal pain/right upper quadrant tenderness	US, CT, MR/ 10 cm/right lobe	Right hepatectomy with infiltrating diaphragm resection	Endometrioid adenocarcinoma	Chemotherapy	Ongoing

*HRT* hormonal replacement therapy; *HY* + *BSO* hysterectomy + bilateral salpingo-oophorectomy; *ESS* endometrial stromal sarcoma; *NA* not applicable

Three criteria were used to diagnose malignant transformation in endometriosis [11]: (1) normal endometrial tissue is found adjacent to malignant tissue, (2) the histopathology of malignant disease should be of endometrial origin, and (3) no other primary tumor can be found. In many cases, including this report, satisfying the first criterion is difficult. Even after extensive sampling, nonneoplastic endometriosis was not observed, presumably because the tissue had atrophied or been destroyed by the carcinoma. Most extraovarian malignant transformations are endometrioid adenocarcinomas, but other histological types, including sarcoma, clear cell carcinoma, and rare cell types, have been reported [5]. Considering the existing 4 cases reported of malignant tumors arising from endometriosis in the liver, one endometrioid adenosquamous carcinoma reported by Weinfeld et al. [13], one adenosarcoma with a benign epithelial component and a malignant mesenchymal component [14], one low-grade endometrial stromal sarcoma [15] and the remaining endometrioid adenocarcinoma [16], as in our case.

Several risk factors have been implicated in the neoplastic transformation of endometriosis [4]. Retrograde menstruation and repeated hemorrhage induce heme and free iron accumulation within endometriotic lesions, resulting in reactive oxygen species formation, which plays a role in carcinogenesis. High levels of inflammatory mediators and activated cytokines/chemokines, as well as aberrant function of almost all types of immune cells in endometriosis promote angiogenesis, cell proliferation, invasion, and metastasis, contributing to both the occurrence and progression of neoplastic processes. Some evidence associates endometriosis with autoimmune diseases. As reported by Mignemi et al., an extremely rare case of nasal localization of endometriosis in a woman affected concurrently by Behcet’s disease, a rare systemic vascular autoimmune disease [18]. Hyperestrogenism, either from exogenous sources or from endogenous overproduction, is also a potential risk factor. However, no definite data indicate the absolute risk of malignant transformation in postmenopausal women using HRT [1, 2]. The patient in this case experienced menopause 4 years prior due to TAH/BSO and did not received any HRT. Heaps et al. [5] reported that only 14 of the 205 cases of malignant tumors arising in endometriosis were associated with known estrogenic stimulation. The possibility of excess estrogen production from fatty tissue could be excluded because our patient was not overweight. Additionally, genetic, and epigenetic factors may contribute to the carcinogenesis of endometriosis [19]. However, we did not perform genetic testing on the patient in this case.

The diagnosis of extrapelvic endometriosis-associated malignancy is challenging [20, 21]. A history of endometriosis and suggestive catamenial symptoms may be contributive, but these do not fit all situations. Tumor markers such as CA125 and CA199 show low specificity; therefore, diagnosis based on these markers is difficult. Although ultrasonography, CT, and MRI are useful, pathognomonic features of extrapelvic endometriosis-associated malignancy have not been described for any of these imaging modalities. Therefore, the gold standard for diagnosis is histologic examination combined with immunochemistry. Moreover, given the heterogeneity of endometriosis-associated malignancy and the potential adverse effects, transhepatic fine needle biopsy or core biopsy may misinterpret the true identity of the lesions. Intraoperative frozen sections should be highly recommended to guide the extent of excision required and to avoid excessive treatment [22]. In this report, the final diagnosis of a malignant tumor arising from hepatic endometriosis was based on the following findings: (1) history of pelvic endometriosis, (2) distinctive histological adenocarcinoma with an epithelial phenotype typical of endometrial origin, and (3) absence of a primary neoplasm found elsewhere.

There are no guidelines on the optimal management of malignant transformation of hepatic endometriosis. Reviewing the reported cases, radical resection has been the primary treatment thus far. No evidence indicates the role of adjuvant therapy for prognosis following surgery. Unlike in this case, no additional treatment was administered in the cases reported by N’Senda et al. [14] and Knowles et al. [16] Collecting more clinical data with long-term follow-up is needed to further understand this type of cancer.

Malignant transformation of hepatic endometriosis is rare and poorly understood complication. No specific biomarkers or sound screening tests currently exist, and accurate diagnosis is primarily based on histopathological evaluation combined with immunohistochemistry staining. Collaborative efforts across multiple disciplines, such as radiology, pathology, oncology, and gynecology, are required when determining the diagnosis and appropriate treatment options for patients with malignant transformation of hepatic endometriosis.

## Supplementary Information

**Additional file 1: Table 1**. Patient characteristics, presentation, and treatment of case reports ofhepatic endometriosis.

## Data Availability

All data related to this case report are available from the corresponding author by request.

## Abbreviations

TAH/BSO: Total abdominal hysterectomy and bilateral salpingo-oophorectomy
CT: Computed tomography
MRI: Magnetic resonance imaging
HRT: Hormone replacement therapy
CA: Carbohydrate antigen
