# Supplementary material for: Malignant transformation of hepatic endometriosis: a case report and literature review
Source: BMC Womens Health. 2021 Jun 21;21:249. doi: 10.1186/s12905-021-01366-6 (PMC8218461; doi:10.1186/s12905-021-01366-6)
Supplement: Supplementary file 1 — Additional file 1: Table 1. Patient characteristics, presentation, and treatment of case reports ofhepatic endometriosis. [file 12905_2021_1366_MOESM1_ESM.docx]

Additional Table 1 ^†^. Patient characteristics, presentation, and treatment of case reports of hepatic endometriosis.

| Author; Year | Age (y) | Menopausal state/HRT | Prior surgery | History of endometriosis | Primary symptom/physical examination | Preoperative diagnosis/ lesion size/location | Treatment |
| --- | --- | --- | --- | --- | --- | --- | --- |
| Finkel,1986 | 21 | Pre/NA | unilateral fallopian tube cyst removal | No | Epigastric pain, nausea, vomiting not related to menses/right upper quadrant tenderness | US, CT/12 cm/left lobe | Cyst enucleation +Danazol therapy |
| Rovati, 1990 | 37 | Pre/No | No | Yes | Chronic, acyclic epigastric pain/epigastric palpable mass | US, CT/ 10cm/left lobe | Left segmentectomy + danazol; Left adnexectomy (second laparotomy for ovarian endometrioma) |
| Verbeke (1), 1996 | 34 | Pre/No | No | No | Acute abdomen/NA | CT/12cm/right lobe | Right hemihepatectomy |
| Verbeke (2), 1996 | 62 | Post/NA | Meckel's diverticulum resection | No | Right epigastric pain | US, CT/12cm/left lobe | excision |
| Cravello, 1996 | 50 | Pre/oral contraceptives for dysmenorrhea | No | Yes | Cyclic right subcostal pain related with menses/No findings | US, CT, MR/ 6cm/right lobe | GnRHa + Conservative tumorectomy with adjacent diaphragm resection |
| Chung,1998 | 40 | Pre/No | Left ovarian cystectomy; | Yes | asymptomatic/No findings | US, CT/6.4 cm/left lobe | Cyst enucleation |
| Inal, 2002 | 25 | Pre/No | No | Yes | Cyclic right subcostal pain related with menses/NA | US, CT, MR/5cm/right lobe | Tru-cut biopsy + danazol |
| Bohra, 2001 | 37 | Pre/No | HY+BSO | Yes | Vague abdominal pain/NA | NA | NA |
| Khan, 2002 | 31 | Pre/Yes | HY+BSO | Yes | Malaise, abdominal distension/ascites and mild jaundice | US, CT/NA/right & left lobe | En bloc cyst resection in the right lobe and left lobe mass was left in situ + intranasal  goserelin |
| Huang, 2002 | 56 | Post/NA | HY+BSO | Yes | Intermittent epigastric pain unrelated with menses/no findings | US, CT, MR/9cm/left lobe | Left hepatectomy |
| Jeanes, 2002 | 31 | Pre/No | Left cystectomy+right oophorectomy; hysterectomy+ left oophorectomy | Yes | Malaise cachexia, abdominal distenstion/jaundice, weight loss | US, CT, MR/12cm/ right lobe; 5.5cm/left lobe | Enucleation in the right lobe and left lobe mass was left in situ + intranasal  goserelin |
| Reid, 2003 | 46 | Post/No | Bladder endometriotic nodule removal; subtotal HY+bilateral oophorectomy+bowel endometriosis resection/Yes | Yes | Right upper quadrant pain/NA | US, CT/11cm/right lobe | Right hemi-hepatectomy, cholecystectomy and diaphragmatic resection |
| Tuech, 2003 | 42 | Pre/No | No | No | Chronic, acyclic epigastric pain/right upper quadrant tender mass | CT/24cm/right lobe | Complete cyst excision |
| Groves, 2003 | 52 | Post/No | HY+ oophrectomy | NA | Right upper quadrant pain/NA | US, CT, MR/multiple lesions, maximum 12cm/right lobe | Right hemihepatectomy |
| Carbone, 2004 | 38 | Pre/No | Caesarean delivery; | No | Irregular upper abdominal pain/right hypochondrial tender mass | CT/13cm/segments II, III and IV | Cyst-pericystectomy |
| Nezat (1), 2005 | 36 | Pre/No | No | Yes | Cyclic epigastric pain/no findings | US,CT/3cm/right lobe | Laparoscopic cyst resection |
| Nezat (2),2005 | 30 | Pre/No | No | Yes | Chronic pelvic pain, dysmenorrhea and painful bowel movements/no findings | US, CT/No preoperative pathological findings | Laparoscopic cyst resection, and resection of coexisting diaphragmatic and pelvic implants |
| Girlanda, 2005 | 34 | Pre/Yes | Sigmoid colon and abdominal wall endometriosis resection; right oophorectomy; total HY+ left salpingo-oophorectomy | Yes | Recurrent right upper quadrant abdominal pain/NA | US, CT, MR/7cm and 6.5 cm perihepatic masses/right lobe | Cyst resection; omental and visceral peritoneal endometriosis excision |
| Lolis, 2007 | 38 | Pre/NA | No | Yes | Recurrent right flank pain/right upper quadrant palpable mass | US, CT/8cm/segment VI | Cyst resection |
| Goldsmith, 2009 | 48 | Post/Yes | HY+BSO | Yes | Chronic right upper quadrant pain/right upper quadrant tenderness | US, MRI/11cm/segment IV, VIII, II and III | Nonanatomical  resection |
| Arshan, 2010 | 61 | Post/NA | HY+ right salpingo-oophorectomy; bowel loop resection | Yes | Epigastric pain/NA | CT/NA/bilobar | Percutaneous true-cut liver biopsy |
| Schuld, 2011 | 39 | Pre/No | No | No | Irregular right upper abdominal pain, hemoptysis, chest pain, cough/ | Bronchoscopy, MR/NA | Atypical resection of segment VIII, transdiaphragmatic pulmonary wedge resection |
| Roesch-Dietlen, 2011 | 25 | Pre/No | No | No | Relapsing right upper quadrant pain/slight right upper quadrant tenderness | US/ multiple small  size gallstones, no other findings | laparoscopic cholecystectomy, biopsy of hemorrhagic area on the surface of the right liver lobe + danazol |
| Rivkine, 2012 | 51 | Pre/NA | HY | No | Epigastric pain unrelated to menses/epigastric pain without palpable mass | US, CT, MR/8cm/segments II and III | Anatomical resection including left lobectomy with diaphragm resection |
| Fluegen, 2013 | 32 | Pre/No | Laparoscopic deroofing of inhepatic cyst and cholecystectomy | No | Constant right upper quadrant abdominal pain/ upper quadrant tenderness, jaundice | MR/12cm/segment IV, V and VIII | Ultrasonic pericysectomy |
| Hertel, 2013 | 44 | Pre/NA | Partial HY+ oophorectomy; surgical repair of incision hernia | No | Sudden onset of severe upper abdominal pain | MR/11.2cm/right lobe | Partial hepatectomy |
| Bouras, 2013 | 35 | Pre/No | No | No | Recurrent epigastric pain/tender, epigastric mass | CT, MR/10cm/left lobe involved right ventricle and diaphragm | left lateral hepatic  sectionectomy, partial right ventricle free wall resection  and medial diaphragm resection |
| Hsu(1), 2014 | 73 | Post/NA | HY | Yes | abdominal pain, dyspnea/NA | NA/7cm/segment VII, extracapsular  posterior to the  liver | Partial hepatectomy |
| Hsu(2), 2014 | 30 | Pre/No | No | Yes | Right abdominal pain, infertility/NA | NA/2cm/Segment VIII | Partial hepatectomy |
| Hsu(3), 2014 | 41 | Pre/NA | HY+ endometriosis covering the whole abdomen | Yes | NA/left upper quadrant palpable mass | CT/17cm/left lobe | Partial hepatectomy |
| Hsu(4), 2014 | 56 | Post/NA | HY+ BSO | Yes | Intermittent epigastric  Pain/NA | NA/9cm/left lobe | Partial hepatectomy |
| Hsu(5), 2014 | 34 | Pre/Yes | HY+ oophorectomy; bowel resection | Yes | Right upper  abdominal pain/NA | MR/6.5cm/ Segment VI,  predominantly  extracapsular  inferior to the  liver | Cyst removal |
| Zhao, 2014 | 36 | Pre/No | Appendectomy; Cesarean section; | NA | Asymptomatic/No findings | CT/6cm/segment IV and V | Cyst resection |
| Sopha, 2015 | 47 | Pre/NA | Right salpingo-oophorectomy; supracervical HY; cesarean section/Yes | No | Right upper quadrant abdominal pain, nausea, vomiting/NA | CT/1.3cm/segment VII | laparoscopic wedge resection |
| Liu, 2015 | 36 | Pre/No | No | No | Right quadrant pain/No findings | US, CT/6.5cm/segment III | pericystectomy |
| Riggi, 2016 | 27 | Pre/No | No | No | Asymptomatic/upper and left abdominal quadrants tenderness | US, CT, MR/30cm/segments IV, V and VII | Left hepatectomy |
| Sherif, 2016 | 44 | Pre/NA | HY; cholecystectomy | Yes | Periodic right upper quadrant pain, vomiting | US, CT, MR/3cm/right lobe | Hepatic segmentectomy |
| Keramidaris, 2018 | 40 | Pre/NA | No | No | Asymptomatic/no findings | US, MR/10.3cm/segments IV, II and III | Laparoscopic cyst resection |
| Rana, 2019 | 42 | Pre/NA | HY + left oophorectomy | No | Episodic, severe right upper quadrant pain, nausea, vomiting/right upper quadrant tenderness | CT/4cm/left lobe | laparoscopic left partial hepatectomy (segment  II and partial segment III) |

^†^ 4 cases of malignant transformation of hepatic endometriosis not included in this table

HRT, hormonal replacement therapy; HY, hysterectomy; BSO: bilateral salpingo-oophorectomy; NA, not applicabl
